# Study on Cardiotoxicity and Mechanism of “Fuzi” Extracts Based on Metabonomics

**DOI:** 10.3390/ijms19113506

**Published:** 2018-11-07

**Authors:** Guangyao Huang, Liang Yang, Wei Zhou, Xianglin Tang, Yuguang Wang, Zengchun Ma, Shan Gao, Yue Gao

**Affiliations:** 1 Department of Pharmacology, Basic Medical College, Anhui Medical University, Hefei 230032, China; huangguangyao@stu.ahmu.edu.cn (G.H.); gaoshan@ahmu.edu.cn (S.G.); 2 Department of Pharmacology and Toxicology, Beijing Institute of Radiation Medicine, Beijing 100850, China; jkliang_yang@126.com (L.Y.); zhouweisyl802@163.com (W.Z.); tangxianglin@139.com (X.T.); wyg79@163.com (Y.W.); mazchun@139.com (Z.M.)

**Keywords:** Fuzi, cardiotoxicity, metabolomics, PI3K/Akt/mTOR, caspase-3

## Abstract

To investigate the toxicity of water and ethanol “Fuzi” (FZ) extracts and to explore the toxicity mechanism in rats. Water and ethanol extracts were prepared. Three groups of rats received the water extract, ethanol extract, or water by oral gavage for seven days. Pathological section staining of heart tissue. Colorimetric analysis was used to determine serum lactate dehydrogenase. The metabolic expression of small molecules in rats was measured by a metabolomics method. Western blotting was used to detect the expression of phosphoinositide 3-kinase (PI3K), protein kinase B (Akt), mammalian target of rapamycin (mTOR), transforming growth factor-β1 (TGF-β1), and caspase-3. Immunohistochemistry was used to detect the expression of CTnI, mTOR, and TGF-β1. The water and ethanol FZ extracts exert cardiotoxic effects via activating the PI3K/Akt/mTOR signaling pathway to induce cardiomyocyte apoptosis.

## 1. Introduction

Traditional Chinese medicine (TCM) is an ancient system of medicine which is still widely used in China and surrounding areas as a complementary and alternative medicine. Despite long clinical practice, the effectiveness and beneficial contribution to public health and disease control of TCM has not been fully established [1,2]. *Aconitum carmichaelii Debx*, the common name of the plants in the genus *Aconitum* L. (Ranunculaceae), are well known worldwide, both for their wide application in the traditional medicine of China, Japan, and Korea. As a widely used Chinese herbal medicine, the roots of *Aconitum carmichaelii Debx* have a wide range of pharmacological effects, commonly applied for various diseases, such as collapse, syncope, rheumatic fever, painful joints, gastroenteritis, diarrhea, oedema, bronchial asthma, various tumors, and some endocrinal disorders like irregular menstruation [3,4]. In Chinese Pharmacopoeia (CP) 2015, *Aconitum carmichaelii Debx* are recorded, extensively distributed in the Sichuan Province of China. The mother root of *Aconitum carmichaelii Debx* is named ‘‘Chuanwu’’, while the daughter or lateral root of *Aconitum carmichaelii Debx* is ‘‘Fuzi’’ (FZ). FZ has commonly been used as an analgesic, anti-inflammatory, and antitumor agent in TCM for 2000 years [5]. However, the toxicity of FZ leads to a potentially high risk of severe problems that are sometimes even life-threatening [6], and its high toxicity has caused many fatal poisonings including accidental, suicidal, and homicidal cases [7]. Cardiotoxicity and neurotoxicity are the main toxic effects caused by FZ. Moreover, death may occur from ventricular arrhythmia within the first 24 h after improper intake of Fuzi [8]. The typical symptoms of FZ poisoning include palpitation, vomiting, nausea, arrhythmia, shock, dizziness, hypotension, and coma [3]. At present, a comparison of the metabolic profiles revealed that four major groups of alkaloids—monoester-diterpene alkaloids (MDAs), amine-diterpenoid alkaloids, diester-diterpene alkaloids (DDAs), and lipoalkaloids—were found in FZ [9]. The toxicity of Aconitum mainly derives from the DDAs, including aconitine, mesaconitine, and hypaconitine [7]. It has been shown that hydrolysates obtained from FZ can be modified into corresponding nontoxic MDAs such as benzoylaconitine, benzoylmesaeonitine, and benzoylhypaconitine [10]. As we know, TCM’s herbal processing approaches, namely “*Paozhi*”, by means of the transformation of secondary plant metabolites, help to reduce the toxicity of the drug, and might exert a large maximal therapeutic efficacy with minimal adverse effects [11]. As a traditional processing form, *Paozhi* could remarkably reduce the toxicity of the FZ by decomposing the DDAs to the relatively lower toxic MDAs [12]. All of the processed FZ must be used. According to the ways of the processing form, it is converted into FZ preparations, including Yanfuzi, Heishunpian, and Baifupian, which are recorded in CP 2015. The traditional processing method involves salting, washing, and soaking the roots. Boiling, peeling, slicing, foaming, cooking, and bleaching reduce cardiotoxicity [13]. However, processing can remove up to 80% of the essential bioactive components required for successful treatment. Consequently, the clinical applications of FZ are limited and careful processing is required to reduce the amount of toxic compounds in the extracts. Different processing, extraction, and decoction methods produce extracts containing different medicinal components of FZ, resulting in differences in toxicity. Extraction with different solvents alters the type and quantity of alkaloids in the extract. The active toxic alkaloids are liposoluble; thus, the DDA content in nonpolar solvent extracts is higher than that in aqueous solvent extracts. At present, there are few studies on the extraction of FZ alkaloids, most of which focus on extraction using boiling water or soaking, and extraction using alcoholic solvents [14].

There is no effective method for evaluating the cardiotoxicity of TCM, and it is still not known how the support vector machines model can be applied to predict the cardiotoxicity in TCM [15]. However, disruption of the phosphoinositide 3-kinase (PI3K)-protein kinase B (Akt)-mammalian target of rapamycin (mTOR) signaling pathway has been linked to cardiovascular disease; thus, investigating the effects of FZ extracts on this pathway may clarify the mechanism of cardiotoxicity. Akt has become a focus of research as a proto-oncogene because it regulates various cell functions, including metabolism, growth, and proliferation, and it plays important roles in survival, transcription, and protein synthesis. Factors that activate Akt cascade amplification include receptor tyrosine kinases, integrin B lymphocyte and T lymphocyte receptors, cytokine receptors, G protein-coupled receptors, and other stimuli that can induce the production of phosphatidylinositol by PI3K [16]. PI3K/Akt activity plays a central role in cellular processes, particularly cell growth, proliferation, and survival [17]. PI3K/Akt signaling pathway disorders are found in a variety of human diseases, including cancer, diabetes, cardiovascular disease, and neuropathy [18]. mTOR is an atypical serine/threonine kinase found in mTOR complex 1 and mTOR complex 2. The PI3K/Akt signaling pathway regulates mTOR activity, and thus regulates cell growth, apoptosis, and autophagy downstream [19]. mTOR signal transduction is abnormal in many diseases, such as cancer, cardiovascular disease, and diabetes [20], and many experiments have shown that PI3K/Akt/mTOR signaling pathway is directly related to cardiac injury and cardiomyocyte apoptosis. It is reported that PI3K/AKT/mTOR signaling pathway may lead to myocardial injury and apoptosis after being stimulated by external factors [21,22]. Among them, Jing Wu et al. [21] have reported that by inhibiting PI3K/AKT/mTOR signal transduction pathway, myocardial injury can be effectively improved. Yihui Yu et al. [22] have also reported that decreasing the expression of PI3K/Akt/mTOR can improve cardiac hypertrophy and fibrosis and decrease cardiomyocyte apoptosis. It is also reported that activation of mTOR may increase apoptosis [23]. Transforming growth factor-β1 (TGF-β1) is mainly produced by cardiac myofibroblasts and fibroblasts and contributes to cardiac fibrosis development, hypertrophy, and apoptosis [24]. TGF-β1 gene expression is increased in the left ventricular myocardium of patients with idiopathic hypertrophic cardiomyopathy or dilated cardiomyopathy and in animals after myocardial infarction [25]. Both the intrinsic and extrinsic apoptotic cell-death pathways are induced in ventricular cardiomyocytes after myocardial ischemia/reperfusion and these pathways converge in the activation of caspase-3 [16,26].

Metabolomics measures the ensemble of small molecules (molecular mass < 2000) in a biological sample. The detailed metabolite profiling of thousands of the secondary metabolites has great potential for directly elucidating plant metabolic processes [27]. It has brought enormous opportunities for improved detection and discovery of biomarkers, and adopts a ‘top-down’ strategy to reflect the function of organisms from terminal symptoms of the metabolic network and understand metabolic changes of a complete system caused by interventions in a holistic context [28,29,30]. This information can be used to evaluate drug efficacy, predict drug toxicity, and diagnose disease [31]. Moreover, drug targets or receptors that are involved in toxic effects can be found through identifying terminal metabolites. A clear understanding of the differentiation mechanism of FZ is essential to evaluate drug safety and for the safe use in the clinic. However, the research is still limited for content changes of several alkaloids to determine its efficacy/toxicity, while ignoring the crude and processed products as a complex group of plant metabolites [12]. Moreover, little is known about the global change of the metabolome of FZ extracts. With the development of new analytical techniques, metabolomics as a research platform could provide comprehensive, detailed, reliable evidence for further study on efficacy/toxicity of processed FZ.

In this study, we investigated the toxicity of FZ extracts with different solvents, and we used metabolomics to detect the metabolic expression of small molecules in rats to clarify the toxicity mechanism of FZ extracts.

## 2. Results

### 2.1. Cardiotoxicity of Water and Ethanol Extracts of FZ

**Figure 1:**
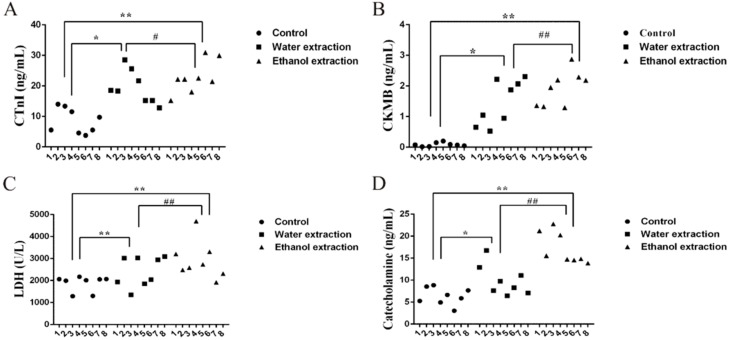
CTnI, CK-MB, LDH, and catecholamine levels in serum measured by ELISA and colorimetric analysis. (**A**) Representative figure of serum CTnI content. (**B**) Representative figure of serum CK-MB content. (**C**) Representative figure of serum LDH content. (**D**) Representative figure of serum catecholamine content. Data are expressed as mean ± SD, *n* = 8. * *p* < 0.05, ** *p* < 0.01 vs. control group; ^#^ *p* < 0.05, ^##^ *p* < 0.01 vs. water extraction group.

**Figure 2:**
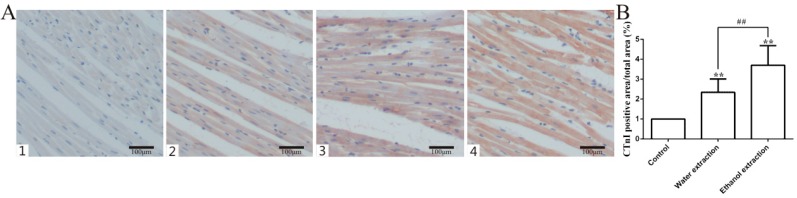
CTnI expression levels in the heart tissues in control, water extraction, and ethanol extraction groups. (**A**) Representative figure of CTnI immunohistological staining in heart (200×); 1: Negative group; 2: Control group; 3: Water extract group; 4: Ethanol extract group. (**B**) Statistic results of CTnI immunohistological staining (*n* = 8); data are expressed as mean ± SD. ** *p* < 0.01 vs. control group; ^##^ *p* < 0.01 vs. water extraction group.

Cardiac troponin (CTnI) and creatine kinase mb isoenzyme (CK-MB) are often measured to detect cardiac injury [32]. The ELISA results for these two indicators in serum are shown in Figure 1A,B. Compared with the control group, the rats treated with water and ethanol extracts of FZ showed heart damage after 7 days (*p* < 0.05). Moreover, the degree of myocardial injury in the ethanol extract group was higher than that in the water extract group (*p* < 0.05). The lactate dehydrogenase (LDH) results were consistent with the CTnI and CK-MB results. Catecholamine is an indicator of cardiac function that induces pleomorphic ventricular tachycardia [33]. Both extracts were cardiotoxic in rats, and the toxicity of the ethanol extract was higher than that of the water extract (Figure 1C). Myocardial injury releases CTnI from myocardial cells; CTnI and catecholamine were detected by immunohistochemistry (Figure 2D,E), which was consistent with the ELISA results.

### 2.2. Pathology Results

**Figure 3:**
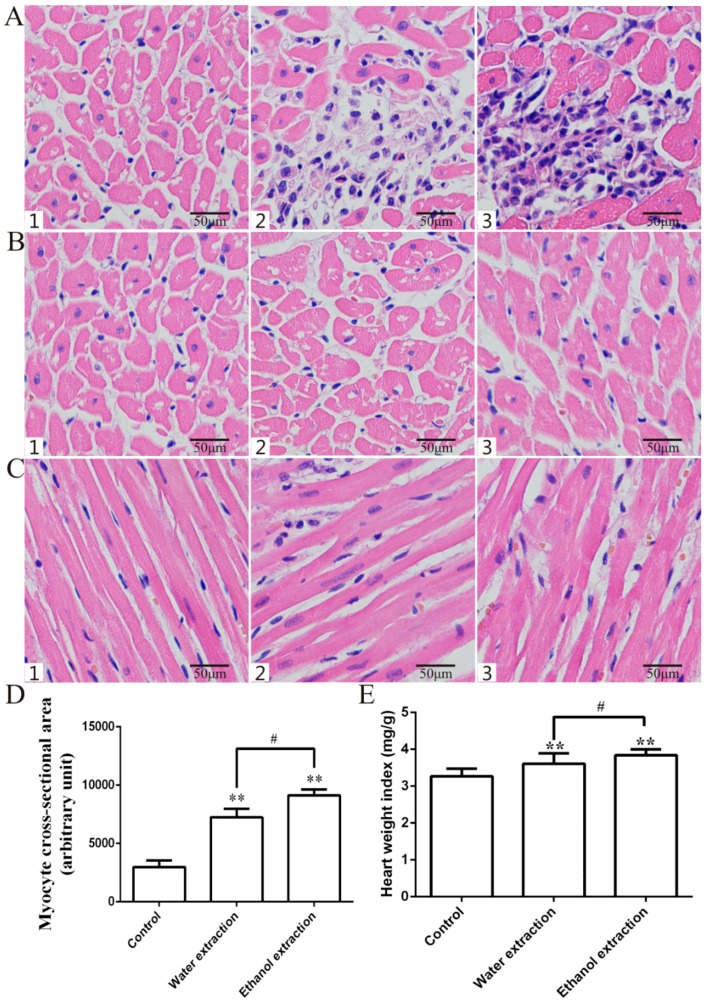
Histopathological examination of heart, HW/BW, and myocyte CSA. (**A**,**B**) Representative figure of myocyte cross-section (hematoxylin-eosin staining (HE) staining, 400×); (**D**) Statistic results of myocyte CSA. 1: Control group; 2: Water extraction group; 3: Ethanol extraction group; (**C**) Representative figure of myocyte long axis (HE staining, 400×); (**E**) Statistic results of HW/BW; data are expressed as mean ± SD, *n* = 8. ** *p* < 0.01 vs. control group; ^#^ *p* < 0.05, vs. water extraction group.

**Figure 4:**
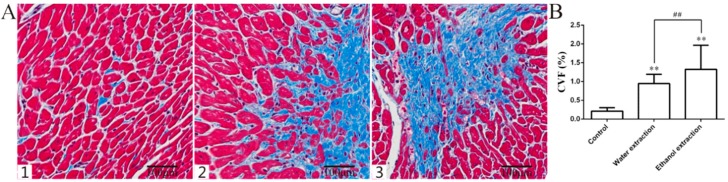
Masson’s trichrome staining of heart tissue and CVF. (**A**) Representative figure of myocardial fibrosis (Masson staining, 200×); (**B**) Statistic results of myocardial fibrosis (*n* = 8); 1: Control group; 2: Water extraction group; 3: Ethanol extraction group; data are expressed as mean ± SD. ** *p* <0.01 vs. control group; ^##^ *p* < 0.01 vs. water extraction group.

The cardiac sections from the three groups showed clear pathological changes in the two groups of rats treated with FZ extracts. Inflammatory infiltration and cardiomyocyte hypertrophy were observed and Masson’s trichrome staining showed fibrosis in the myocardial tissue of the two groups. Cardiac histology showed that the myocyte cross-sectional area (CSA) and collagen volume fraction (CVF) in the cardiac myocytes of the two treated groups were significantly higher than those in the control group, and that the CSA and CVF in the ethanol extract group were higher than those in the water extract group (Figure 3A–D and Figure 4) (*p* < 0.05). Compared with the control group, the heart weight index (heart weight/body weight, HW/BW) was significantly higher in the two groups treated with FZ extracts (Figure 3E) (*p* < 0.05).

### 2.3. Identification of Differential Metabolites

**Figure 5:**
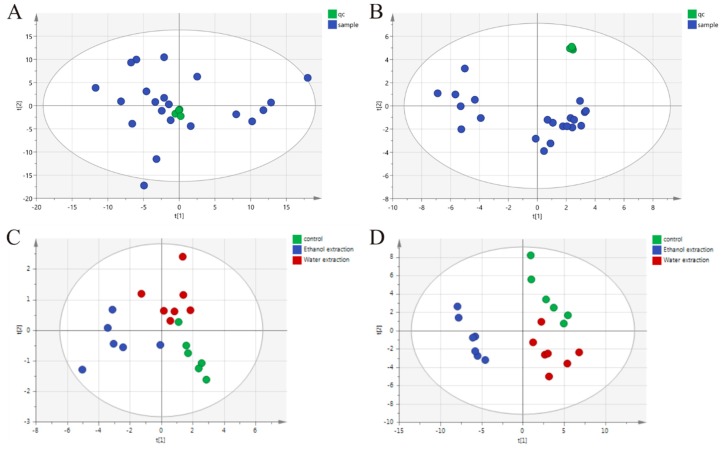
Multiple pattern recognition of the serum metabolites in the control, water extract, and ethanol extract groups on day 7. (**A**) QC of reversed phase C18 separation. (**B**) QC of hydrophilic chromatogram separation. (**C**) Representative figure of reverse phase C18 column detection chart. (**D**) Representative figure of hydrophilic chromatogram. Control group (green), ethanol extraction group (blue), water extraction group (red).

**Figure 6:**
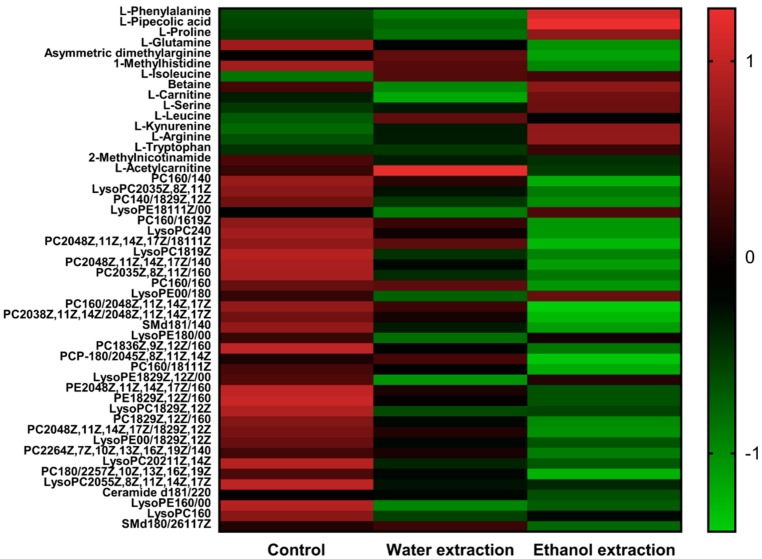
Serum metabolites in the control, water extract, and ethanol extract groups. The concentration of small molecule metabolites in each group of rats was measured and treatment was normalized, and the −1 to +1 range was used to represent the contrast intensity; With reference to the ruler, the depth of the color represents the level of the content.

Based on the metabolic differences among the three groups, we used principal component analysis (PCA) to classify the differential metabolic phenotypes. In this result, the model interpretation rate and the model prediction rate of reversed phase C18 separation partial model are R^2^X (model interpretation rate) = 0.672, Q^2^ (model prediction rate) = 0.517. Hydrophilic chromatogram separation partial detection results are R^2^X = 0.771, Q^2^ = 0.584. The quality control (QC) results show that in reversed phase C18 separation, phosphocholine(20:4(8Z,11Z,14Z,17Z)/14:0) is an relative standard deviation of 5.8 out of four QC. QC and all samples were used to make PCA, to find QC together. The RSD of the four QC in the L-Phenylalanine in hydrophilic chromatogram separation is 1.75 and the QC is also gathered together (Figure 5A,B). It shows that the stability of the whole process can be guaranteed. PCA scores showed that the three groups were clustered (Figure 5C,D), and the top 50 significant ions were selected for metabolite identification (Figure 6). The 50 compounds were screened by VIP (Variable Importance in the Projection) (threshold > 1) of OPLS-DA (orthogonal projection to latent structures discriminant analysis) model and combined with P value of *t*-test (*p* < 0.05) to search for differentially expressed metabolites, and the 50 metabolites were detected in all three groups of rats. More than 11 significantly altered metabolites were detected in the three groups of rats (Table 1). The 11 significantly altered metabolites refers to comparison with the control group; there were 11 metabolites with significant changes in the ethanol extract group and water extract group of FZ. The serum samples from the rats treated with the water extract showed 11 significantly altered metabolites and those from the rats treated with the ethanol extract showed 9 (Table 1). Leucine, isoleucine, tryptophan, kynurenine, arginine, and serine levels were elevated in the water extract group and the ethanol extract group. The increase in leucine in the ethanol extract group was higher than that in the water extract group. Betaine and methylhistidine were decreased in the treated groups.

### 2.4. Cardiotoxicity of FZ Extracts in Rats via the PI3K/Akt/mTOR Signaling Pathway

**Figure 7:**
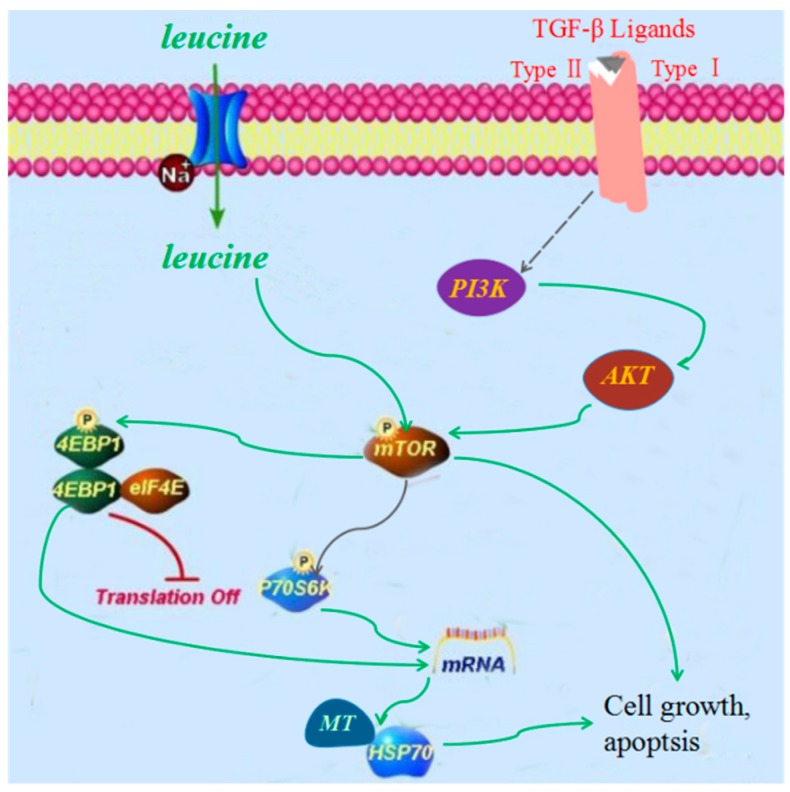
Molecular and protein network function. The solid arrows: Direct Stimulatory Modification; the dotted arrow: Tentative Stimulatory Modification; TGF-β: transforming growth factor-β; AKT: protein kinase B; MT: metallothionein; HSP70: heatshockprotein70; P: phosphorylation.

**Figure 8:**
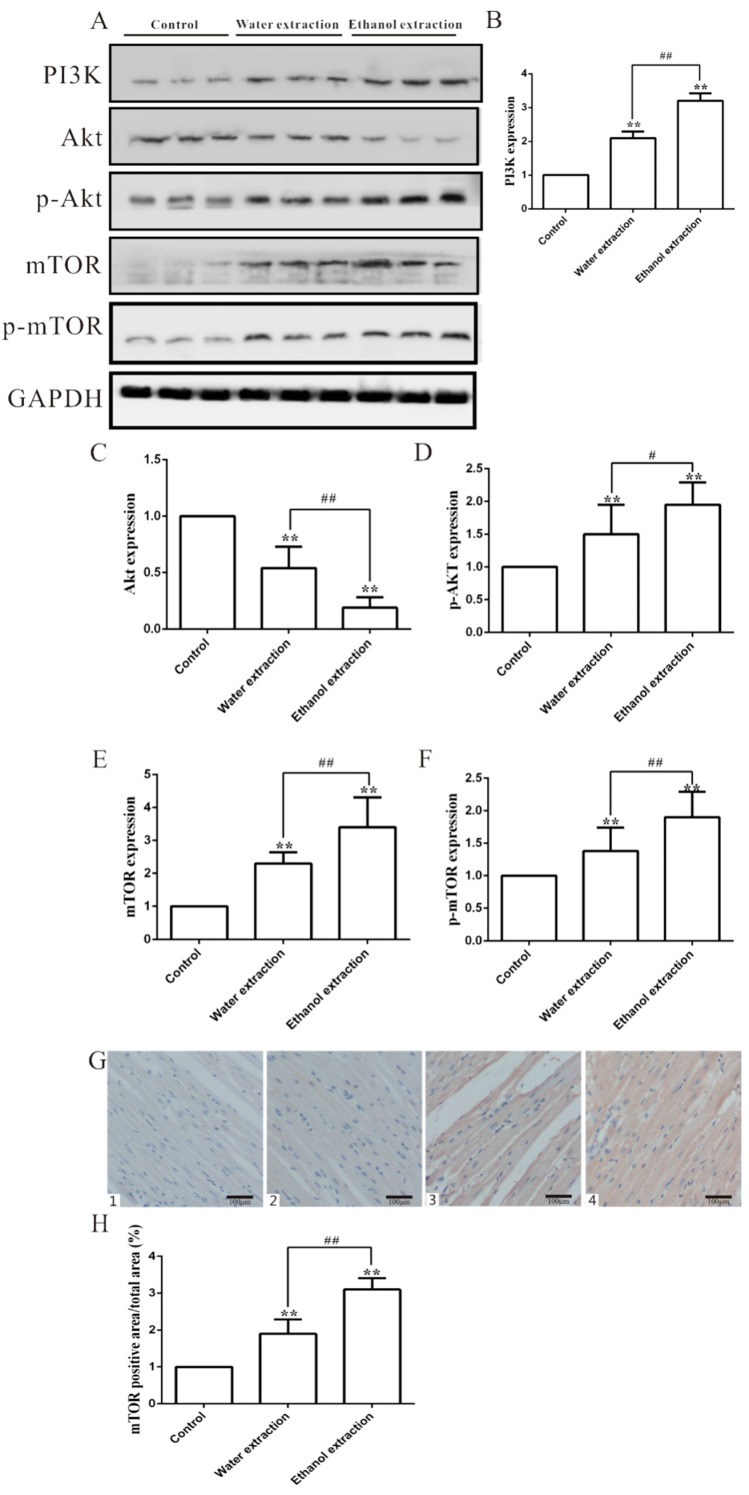
The PI3K/Akt/mTOR expression levels in the heart tissues in control, water extraction, and ethanol extraction groups. (**A**) PI3K, Akt, p-Akt, mTOR, and p-mTOR protein expression levels; (**B**–**F**) Quantitative analyses of PI3K, Akt, p-Akt, mTOR, and p-mTOR protein (*n* = 3); (**G**) Representative figure of mTOR immunohistological staining in heart (200×); 1: Negative group; 2: Control group; 3: Water extract group; 4: Ethanol extract group; (**H**) Statistic results of mTOR immunohistological staining (*n* = 8); GAPDH: glyceraldehyde-3-phosphate dehydrogenase; The protein expression levels were determined by Western blot analyses and relative band intensities were analyzed by the Image J software; data are expressed as mean ± SD. ** *p* < 0.01 vs. control group; ^#^ *p* < 0.05, ^##^ *p* < 0.01 vs. water extraction group.

Leucine upregulation may activate mTOR signaling [19], and excessive activation or inhibition of mTOR signaling can induce cardiac disorders [20]. Therefore, we speculated that the following two mTOR signaling pathways may be involved in FZ-induced cardiotoxicity. mTOR affects cell growth and apoptosis [34], but it is also regulated by the upstream PI3K/Akt signaling pathway [35]. The tumor necrosis factor-β (TNF-β) pathway stimulates PI3K activity. TGF-β1 can induce significant cardiovascular adverse effects, such as cardiac arrhythmias [36] and cardiac valve abnormalities [37]. The predicated molecular-function network may be responsible for the toxicity-reducing effect of processing FZ (Figure 7). The PI3K/Akt/mTOR signaling pathway is closely related to apoptosis and autophagy [31,35]. Compared with the control group, the protein expression of PI3K, p-Akt (AKT phosphorylation), mTOR, and p-mTOR (mTOR phosphorylation) in myocardial tissue of rats treated with FZ extracts was significantly upregulated, which reflected the increase in cardiomyocyte apoptosis. The expression of related proteins in the ethanol extract group was higher than that in the water extract group (Figure 8A–F). Furthermore, the levels of mTOR in myocardial tissue detected by immunohistochemistry were also higher in the ethanol extract group than in the water extract group (Figure 8G,H).

### 2.5. Water and Ethanol Extracts of FZ Induce Cardiomyocyte Apoptosis in Rats

**Figure 9:**
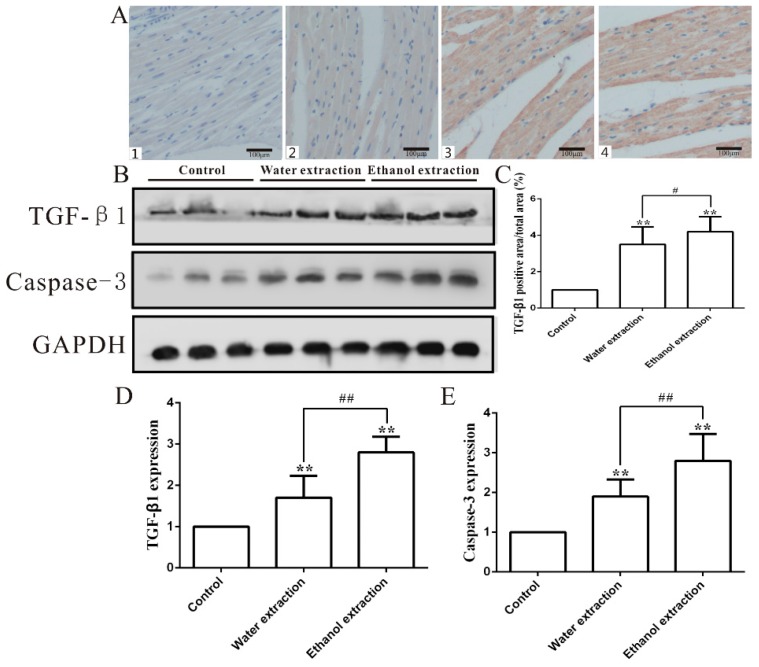
The TGF-β1 and caspase-3 expression levels in the heart tissues in control, water extraction, and ethanol extraction groups. (**A**) Representative figure of TGF-β1 immunohistological staining in heart (200×); 1: Negative group; 2: Control group; 3: Water extract group; 4: Ethanol extract group; (**B**) TGF-β1 and caspase-3 protein expression levels; (**C**) Statistic results of TGF-β1 immunohistological staining (*n* = 8); (**D**,**E**) Quantitative analyses of TGF-β1 and caspase-3 protein (*n* = 3); the protein expression levels were determined by Western blot analyses and relative band intensities were analyzed by the Image J software (a common image processing software); data are expressed as mean ± SD. ** *p* < 0.01 vs. control group; ^#^ *p* < 0.05, ^##^ *p* < 0.01 vs. water extraction group.

Upregulation of TGF-β1 expression can cause adverse cardiac effects [38]. Thus, we speculated that activating the TGF-β signaling pathway contributed to FZ-induced cardiotoxicity. The level of TGF-β1, which is the critical protein in TGF-β signaling, in the myocardial tissue of rats treated with FZ extracts was significantly increased, and the TGF-β1 expression in the myocardial tissue of the ethanol extract group was higher than that of the water extract group (Figure 9B,D). The levels of TGF-β1 in myocardial tissue detected by immunohistochemistry were consistent with those detected by Western blotting (Figure 9A,C). Caspase-3 plays a key role in apoptosis [16]. The expression of caspase-3 was significantly increased in the myocardium of the rats treated with FZ extracts. The increase in the ethanol extract group was higher than that in the water extract group and control group (Figure 9B,E).

## 3. Discussion

FZ is a common Chinese medicine that has been widely used in the treatment of many diseases [13]. DDAs are the main components of FZ, which exert anti-inflammatory, analgesic, and neuromuscular blocking activities [36,39]. These diterpenoid alkaloids can also cause arrhythmia, respiratory failure, and other toxic side effects [40]. We examined the toxicity of water and ethanol FZ extracts in rats and the mechanism of toxicity. The profile of toxic chemical constituents may vary between extracts; thus, the toxicities of herbal preparations may vary between different animals, even of the same species. It is important to determine whether the extracts or the animals cause the differences in toxicity. In this work, we gave rats water or ethanol FZ extracts for 7 days, and found that both extracts caused heart damage. Other studies have also shown that FZ extracts are cardiotoxic [41]. In another study, our team explored the components of FZ water and ethanol extracts. Moreover, the DDAs were quantified [42]. The differences in the metabolism of small molecules between the control group and the rats given FZ extracts showed that the extracts affected the metabolism, with different effects on the organs. There were significant metabolic differences between the water and ethanol FZ extracts in vivo.

According to TCM, processing can reduce the toxicity of herbs, and this theory has been supported by modern studies [40]. Our results for CTnI, CK-MB, and LDH levels indicated that the rats given the FZ extracts suffered heart damage, and the heart damage in the ethanol extract group was the greatest. Performance was consistent among these three indicators of heart injury. This result indicates both extraction methods produce cardiotoxic extracts, but that the toxicity is different. This finding is also reflected in the effects observed in the heart tissue. The immunohistochemistry results for CTnI in heart tissue showed that the release of cardiac CTnI was increased, which indicated that myocardial injury occurred. There was obvious inflammatory infiltration in the myocardial tissue of two groups treated with FZ extracts. Compared with the control group, the cardiac myocytes of the treated groups were hypertrophic, and the hypertrophy was greatest in the ethanol extract group. This suggests that FZ is cardiotoxic in rats, although this toxic dose does not cause serious heart damage or death, due to compensatory mechanisms. The rats responded to the external toxic injury via autogenic hypertrophy to compensate for the function of the apoptotic cardiomyocytes. The greater the toxicity, the greater the compensation was [39]. The two groups of rats given FZ extracts all had myocardial fibrosis, which further indicated that the FZ extracts were cardiotoxic, and the toxicity of the ethanol extract of FZ was greater than that of the water extract.

There were significant differences in more than 12 metabolites among the three groups of rats. The rats fasted for 24 h before blood sample collection. Exogenous amino acids were eliminated within 24 h and were not detected in the serum. The endogenous amino acids leucine and glutamine showed significant differences among the three groups. Leucine upregulation may activate mTOR signaling [43,44], and excessive activation or inhibition of mTOR signaling could induce cardiac disorders [45,46]. Glutamine plays an important role in cell growth and development, and it also regulates the expression of mTOR [47,48]. The PI3K/Akt/mTOR signaling pathway is required for the prosurvival signaling cascade under a wide variety of circumstances. The PI3K/Akt/mTOR signaling pathway participates in and regulates the apoptosis of cardiomyocytes [49]. PI3K/Akt activity plays a crucial role in various cellular processes, including cell growth, proliferation, and survival [50]. PI3K/Akt activity can also act directly on mTOR, and affect cells by regulating the expression of mTOR [39]. However, the TNF-β signaling pathway also translocates PI3K and activates the PI3K/Akt/mTOR signaling pathway [51]. mTOR, in its nonpathological capacity, amalgamates multiple signals to stimulate growth and survival, increase cellular metabolism, and promote transcription and translation through activating downstream targets. Multiple reports have provided evidence of mTOR dysregulation in a host of diseases, including diabetes, cancer, hypertrophy, and heart failure [52]. Inhibition of mTOR signaling may be closely related to the pathogenesis of cardiac hypertrophy [45], which may cause heart failure, and lead to morbidity and mortality [53,54]. In addition, inhibition of mTOR signaling can induce acute cardiotoxicity [55]. Our Western blot analyses showed that FZ extracts significantly increased the expression of mTOR in myocardial tissue, suggesting that the FZ extracts were toxic to cardiomyocytes through altering mTOR expression. In addition, compared with the control group, the two groups given FZ extracts showed high PI3K, p-Akt, and TGF-β1 expression. These results suggest that FZ extracts activated the PI3K/Akt/mTOR signaling pathway and were toxic to cardiomyocytes. TGF-β1 regulates the growth, development, and apoptosis of cardiomyocytes [37]; thus, FZ extracts may also induce cardiotoxicity through the TNF-β signaling pathway. However, it is not clear whether the extracts activated the PI3K/Akt/mTOR pathway by activating the TNF-β signaling pathway, and this question requires further investigation.

Death receptor- and mitochondria-mediated apoptotic signaling activate caspase-3, which then acts as a terminal effector of apoptosis by cleaving various substrate proteins and also amplifies the death signal from the plasma membrane by activating additional caspases. Many clinical and animal studies have confirmed that inhibition of caspases, such as caspase-3, attenuates cardiac dysfunction and improves survival during the progression to end-stage heart failure [56]. Therefore, caspase-3 plays an important role in cardiac function and apoptosis. FZ extracts induced caspase-3 overexpression, which confirmed that the cardiotoxicity of FZ is caused by cell apoptosis. The caspase-3 expression also indicated that the ethanol extract was more toxic than the water extract. In addition, caspase-3 promotes inflammation [57], and TGF-β is often chronically overexpressed in disease states, such as cancer and inflammation [58]. Thus, we speculate that activation of caspase-3 and the TGF-β pathway contributed to FZ-induced inflammation in heart tissues, which could explain the inflammatory cell infiltration in the hearts of the FZ-treated rats.

In summary, our findings demonstrate that FZ is cardiotoxic in rats. The cardiotoxicity of FZ is produced by activating the PI3K/Akt/mTOR signaling pathway and regulating the TNF-β signaling pathway. FZ also activates caspase-3, inducing inflammation and apoptosis in cardiac myocytes. The significant metabolic differences in small molecules in rats indicated that the toxicity of the ethanol extract was greater than that of the water extract. Moreover, the activation of the signaling pathways of the two extracts was also different. These results demonstrate that the medicinal and toxic compounds vary widely in preparations of the same TCM obtained by different processing, extraction, and decocting methods, reinforcing the need for safe, appropriate, and effective methods of using TCM.

## 4. Materials and Methods

### 4.1. Animals

A total of 24 Sprague–Dawley (SD) male rats (200 ± 20 g) were obtained from Beijing Vital River Laboratory Animal Technology (Beijing, China) Co., Ltd. [certificate no. SCXK (Jing) 2016-0006] and fed in our animal facility. All the animals were handled and used under strict observation of the rules and regulations outlined in the National Institutes of Health Guide for the Care and Use of Laboratory Animals (NIH Publications No. 8023, revision 1978), and approval (IACUC-DWZX-2018-008, April 2018) was granted by the Animal Care and Use Committee, Academy of Military Medical Sciences, Beijing, China. The rats were kept under standard conditions, in solid-bottomed polypropylene cages with a 12 h dark–light cycle and fed commercial rat chow ad libitum. Three days after acclimatization, the rats were given FZ extracts for seven days and samples were collected.

### 4.2. Materials

FZ was obtained from EFEBIO (Shanghai, China). Rabbit polyclonal antibodies against GAPDH (ab181602) and antirabbit immunoglobulin G (IgG) secondary antibody (ab7090) were obtained from Abcam (Beijing, China). PI3K (21890-1-AP) and TGF-β1 (21898-1-AP) were obtained from Proteintech. Akt (#9272), p-Akt (#4060), mTOR (#2983), p-mTOR (#5536), and caspase-3 (#9662) were obtained from Cell Signaling Technology (Beijing, China). The CTnI ELISA kit (E08594r) and the CK-MB ELISA kit (E14403r) were obtained from Cusabio (Beijing, China). Catecholamine ELISA kit (E14403r) was obtained from Shanghai Gu Du Biotechnology (Shanghai, China) Co., Ltd.

### 4.3. Water and Ethanol Extracts of FZ

#### 4.3.1. Water Extracts of FZ

Take 50 g Yanfuzi and use a pulverizer to break FZ to the size of 0.5–1 cm. Place the broken FZ in the beaker and soak in the distilled water of 500 mL for 8 h. Boil the water for 30 min followed by siphoning of the water. Add a further 500 mL of water to the solid and boil for a further 30 min and collect the water. Combine the extracts collected twice. Control the water temperature of the rotary evaporator at 35 °C, and concentrate the extract to 100 mL [59]. The rotary evaporator will only separate the moisture from the extract. Therefore, the concentration of the extractant can be calculated as 0.5 g/mL by the amount of raw medicine. The extract was subsequently stored at −20 °C.

#### 4.3.2. Ethanol Extracts of FZ

Take 50 g Yanfuzi and use a pulverizer to break FZ to the size of 0.5–1 cm. Place the broken FZ in the beaker with 500 mL 80% ethanol solution and seal for 24 h before siphoning the ethanol extract. Add a further 500 mL 80% ethanol solution and again allow to soak for 24 h. Combine both extractions. Control the water temperature of the rotary evaporator at 35 °C, and concentrate the extract to 100 mL. The rotary evaporator will only separate the moisture from the extract. Therefore, the concentration of the extractant can be calculated as 0.5 g/ml by the amount of raw medicine [60]. The extract was subsequently stored at −20 °C.

### 4.4. Grouping and Experimental Design

The animals were randomized into three groups (*n* = 8 per group). Different FZ extracts were given by gavage for seven days. The control group was given distilled water. All animals were fed a regular chow diet and given tap water.

Group 1: Control group. Eight rats were given distilled water for seven days.

Group 2: Water FZ extract group. Water FZ extract was given to eight rats at a dose of 0.35 g/kg for seven days [61].

Group 3: Ethanol FZ extract group. Ethanol FZ extract was given to eight rats at a dose of 0.35 g/kg for seven days.

### 4.5. Serum Sample Preparation

#### 4.5.1. Analysis of Serum Samples by Reversed-Phase Chromatography

The plasma/serum samples were thawed on ice at 4 °C for 30–60 min. An aliquot of plasma/serum (100 µL) was placed in a labeled 1.5 mL microcentrifuge tube. Chloroform/methanol (3:1) was added and the mixture was ultrasonicated for 1 h. Water (100 μL) was added and the mixture was mixed thoroughly, and then centrifuged at 4 °C and 12,000 rpm for 10 min. The subnatant (300 μL) was dried, isopropyl alcohol/acetonitrile (1:1, 400 μL) was added, and the residue was dissolved by ultrasonication. The solution was centrifuged at 12,000 rpm for 10 min, and the supernatant (100 µL) was transferred to a 200 µL vial insert for analysis.

#### 4.5.2. Method of Sample Treatment for Hydrophilic Chromatographic Analysis of Serum

Plasma/serum samples were thawed on ice at 4 °C for 30–60 min. An aliquot (100 µL) of plasma/serum was placed in a labeled 1.5 mL microcentrifuge tube, acetonitrile was added (300 µL). The mixture was mixed on a vortex mixer (P&Q Science, Shanghai, China) for 15 s, and then the protein precipitate was pelletized in a centrifuge (Beckmann, Beijing, China) at 4 °C and 12,000 rpm for 10 min. The supernatant (100 µL) was transferred to a 200 µL vial insert for analysis.

### 4.6. Chromatography

Ultra-high-performance liquid chromatography was performed on a rapid separation liquid chromatography system (Dionex UltiMate 3000, Thermo Fisher Scientific, Beijing, China) with a reversed-phase C18 column or a hydrophilic interaction liquid chromatography column using the gradient conditions shown in Table 2 and Table 3.

#### 4.6.1. Reversed-Phase Chromatographic Separation

For C18 separation, mobile phase A was acetonitrile/water (60:40) and mobile phase B was isopropanol/acetonitrile (90:10). Mobile phases A and B contained 0.1% formic acid and 10 mmol/L ammonium acetate. The gradient conditions for reversed-phase C18 separation are shown in Table 1. The column was an HSS T3 column (2.1 × 100 mm, 1.8 µm, Waters, Beijing, China) operated at 45 °C. The flow rate was 300 µL/min and the injection volume was 1 µL.

#### 4.6.2. Hydrophilic Chromatographic Separation

For hydrophilic interaction liquid chromatography, mobile phase A was acetonitrile and mobile phase B was water. Mobile phases A and B contained 0.1% formic acid and 10 mmol/L ammonium acetate. The column was a BEH Amide column (2.1 × 100 mm, 1.7 µm, Waters, Beijing, China) operated at 40 °C. The flow rate was 300 µL/min and the injection volume was 1 µL.

#### 4.6.3. QC

In order to ensure the reliability of the data, we also tested the sample QC (Quality Control) at the beginning of the test needle, and then every seven samples were tested again to monitor the whole process.

### 4.7. Mass Spectrometry

A hybrid quadrupole Orbitrap mass spectrometer (Q Exactive, Thermo Fisher Scientific, Beijing, China) equipped with a HESI-II probe (Thermo Fisher Scientific, Beijing, China) was used. The heated capillary temperature was 320 °C, the sheath gas pressure was 30 psi, the auxiliary gas setting was 10 psi, the heated vaporizer temperature was 300 °C, and the positive and negative spray voltages were 3.7 and 3.5 kV, respectively. The sheath gas and auxiliary gas were nitrogen. The collision gas was also nitrogen at a pressure of 1.5 mTorr. The parameters for the full mass scan were as follows: a resolution of 70,000; an auto gain control target under 1 × 106; a maximum isolation time of 50 ms; and an mass-to-charge ratio (*m*/*z*) range of 50–1500. The liquid chromatography–mass spectrometry system was controlled using Xcalibur 2.2 SP1.48 software (Thermo Fisher Scientific, Beijing, China), and data were collected and processed with the same software.

### 4.8. Histological and Morphological Analyses of the Heart

Neutral 10% buffered formalin was employed to immersion-fix the heart apex for histological analysis. Hematoxylin-eosin (HE) and Masson’s trichrome staining were used to observe the paraffin sections (5 μm). Subsequently, the CSA and CVF in the digital microscope images were quantitatively analyzed with ImageJ (a common image processing software, National Institutes of Health).

### 4.9. Measurement of Serum CTnI and CK-MB Activity Levels in Serum and Cardiac Tissue

Serum CTnI and CK-MB activities in serum and cardiac tissue were estimated using ELISA according to the manufacturer’s instructions.

### 4.10. Measurement of Myocardial Enzymes

Serum LDH levels were measured to assess myocardial injury. Myocardial enzymes were measured using kits (Nanjing Jiancheng Institute of Biological Engineering, Nanjing, China) according to the manufacturer’s instructions.

### 4.11. Western Blotting of PI3K, Akt, mTOR, TGF-β1, and Caspase-3

Proteins were extracted from frozen heart tissue and the protein concentration was determined by a BCA (bicinchoninic acid) Protein Assay Kit (P0010, Beyotime, Beijing, China). Equal amounts of loaded protein were separated by SDS-PAGE (sodium dodecyl sulfate-polyacrylamide gel electrophoresis) and transferred to polyvinylidene difluoride membranes, which were then blocked at room temperature for 1 h with 5% dry skim milk in Tris-buffered saline containing 1% Tween-20. The membranes were reacted with antibodies for the glucocorticoid receptor, PI3K (1:1000), Akt (1:1000), p-Akt (1:1000), mTOR (1:1000), p-mTOR (1:1000), TGF-β1 (1:1000), and GAPDH (1:1000) overnight at 4 °C. The membranes were washed thoroughly. PI3K, Akt, p-Akt, mTOR, p-mTOR, TGF-β1, and caspase-3 incubated with antirabbit IgG secondary antibody were conjugated to horseradish peroxidase for 1 h (1:5000), and then washed thoroughly. The protein bands were detected by chemiluminescence reagents (ECL kit, Amersham Biosciences, Beijing, China). A digital imaging system (ImageQuant LAS 500, GE Healthcare Life Sciences, Beijing, China) was used to visualize protein bands, and densitometry was performed with ImageJ software. The density of each immunoreactive band was normalized to the density of the corresponding GAPDH band.

### 4.12. Immunohistochemistry of CTnI, mTOR, and TGF-β1

Immunohistochemical staining was performed with an Ultra-Sensitive S-P kit (Bosterbio and Bioss, Beijing, China) according to the manufacturers’ instructions. In 10 mM sodium citrate (pH 6.0), the sections were deparaffinized, and then they received microwave treatment for 10 min twice. At room temperature, the endogenous peroxidase was incubated in endogenous peroxidase blocking solution for 10 min. The rabbit polyclonal antibodies against CTnI (1:100 dilution) and polyclonal antibodies against TGF-β1 and mTOR (1:200 dilution) at 4 °C were used as primary antibodies for 18 h. The sections were washed three times using phosphate-buffered saline (PBS), and then incubated with biotin-conjugated antirabbit secondary antibody for 10 min. The sections were washed three times with PBS, and then treated with streptavidin-peroxidase for 10 min. The sections were washed with PBS three times, subjected to hematoxylin counterstaining, and then incubated in diaminobenzidine for 5 min. Images were recorded with a digital camera (DM IL, DC 300, Leica, Beijing, China) for all sections.

### 4.13. Statistical Analysis

In this study, all results are from at least three independent experiments and data are expressed as the mean ± Standard Deviation (SD). Data were analyzed by one-way analysis of variance (one-way ANOVA) to test the significance of differences between the control and drug-treated groups. Multiple comparison between the groups was performed using Scheffe method. *p* < 0.05 was considered statistically significant.

## Figures and Tables

**Table 1:** Significantly altered metabolites in the serum samples of control, water extract, and ethanol extract rats (*n* = 8).

Number	t_R_ (min)	Extract Mass	Formula	Compound	Fold Change	
					Water Extract vs. Control	Alcohol Extract vs. Control
1	3.2376	204.1224	C_9_H_17_NO_4_	Acetylcarnitine	↓/0.87	↑/1.02
2	4.7173	162.1120	C_7_H_15_NO_3_	Carnitine	↑/1.17	—
3	4.75298	132.1016	C_6_H_13_NO_2_	Isoleucine	↑/1.33	↑/1.35
4	4.82875	205.0966	C_11_H_12_N_2_O_2_	Tryptophan	↑/1.11	↑/1.14
5	4.85995	209.0915	C_10_H_12_N_2_O_3_	Kynurenine	↑/1.18	↑/1.11
6	4.93126	132.1016	C_6_H_13_NO_2_	Leucine	↑/1.21	↑/1.39
7	4.9669	118.0861	C_5_H_11_NO_2_	Betaine	↓/0.96	↓/0.86
8	6.4243	106.0500	C_3_H_7_NO_3_	Serine	↑/1.126	↑/1.01
9	6.7497	203.1498	C_8_H_18_N_4_O_2_	Asymmetric dimethylarginine	↓/0.60	—
10	7.2177	170.0920	C_7_H_11_N_3_O_2_	Methylhistidine	↓/0.67	↓/0.97
11	7.24445	175.1185	C_6_H_14_N_4_O_2_	Arginine	↑/1.39	↑/1.12

t_R_: retention time; ↑: upregulation; ↓: downregulation; —: no significant change.

**Table 2:** The gradient conditions for reversed-phase C18 separation for lipid.

Time (min)	A (v%)	B (v%)
0	80	20
2	70	30
5	55	45
6.5	40	60
12	35	65
14	15	85
17.5	0	100
18	0	100
18.1	80	20
19.5	80	20

**Table 3:** The gradient conditions for hydrophilic separation of polar metabolites.

Time (min)	A (v%)	B (v%)
0	95	5
1	95	5
7	50	50
9	50	50
9.1	95	5
13	95	5

## Data Availability

The authors declare that all data generated and analyzed in the present study are fully available without restriction.
